# Supplementary material for: Enhanced flexural wave sensing by adaptive gradient-index metamaterials
Source: Sci Rep. 2016 Oct 17;6:35048. doi: 10.1038/srep35048 (PMC5066180; doi:10.1038/srep35048)
Supplement: Supplementary Information [file srep35048-s1.pdf]

Supplementary material: Enhanced flexural wave sensing by adaptive gradient-index metamaterials

Y.Y. Chen, R. Zhu, M.V. Barnhart & G.L. Huang[1]

Department of Mechanical and Aerospace Engineering, University of Missouri, Columbia, MO, 65211, USA.

In this supplementary material, flexural wave amplifications along a gradient-index (GRIN) waveguide with slowly varying material parameters is firstly characterized based on the Wentzel-Kramer-Brillouin (WKB) approximation. Then, a theoretical model of flexural waves propagating along the adaptive metamaterial beam considering both piezoelectric and shunting circuit effects is developed. Dispersion relations for the adaptive metamaterial beam with uniform shunting circuits are obtained analytically for the design of the gradient shunting circuits integrated in the final GRIN metamaterial beam used for wave amplification. After that, wave transmission and amplification properties of the metamaterial enhanced sensing system are characterized based on the theoretical model. The amplification effects are finally defined by the voltage gain across the electrodes of each of the constitutive piezoelectric patches.

1. Flexural wave amplifications based on WKB approximation

In order to quantitatively evaluate the flexural wave amplification, the WKB approximation is employed to characterize the propagation of flexural waves along a GRIN beam. In this theoretical approach, the reflections between unit cells of the GRIN metamaterial beam with shunted

[1]Correspondence and requests for materials should be addressed to G.L.H. (email: huangg@missouri.edu).

piezoelectric patches are ignored, and the metamaterial beam is modeled as a GRIN beam with continuously and slowly varying effective bending stiffness.

The governing equation for an inhomogeneous Euler-Bernoulli beam is given by

$$\rho_e(x)A_e(x)\frac{\partial^2 w_e(x,t)}{\partial t^2}+\frac{\partial^2}{\partial x^2}\left(E_e(x)J_e(x)\frac{\partial^2 w_e(x,t)}{\partial x^2}\right)=0, \tag{1}$$

where $w_e(x,t)$ denotes the transverse deflection of the beam, and the mass density ρ_e, cross-sectional area A_e, Young's modulus E_e, and moment of inertia J_e are considered as functions of x along the beam. According to the WKB approximation, the general solution of Eq. (1) can be written as

$$w_e=\hat{w}_e(x)e^{-i\omega t}e^{iS(x)}, \tag{2}$$

where $S=\{\pm 1,\pm i\}\omega^{\frac{1}{2}}\int_{x_0}^{x}\left(\frac{\rho_e A_e}{E_e J_e}\right)^{\frac{1}{4}}dx$ representing two propagated and two evanescent waves, respectively, and $\hat{w}_e$ is the wave amplitude to be determined. If one considers the infinitesimal section of the beam to be homogeneous, the local wavenumber $k(x)$ of this beam section can then be defined by

$$k(x)=\left(\frac{\rho_e A_e\omega^2}{E_e J_e}\right)^{\frac{1}{4}}. \tag{3}$$

In order to avoid the complex mathematical forms involving in the original WKB method, the expression for the amplitude $\hat{w}_e$ will be derived such that no energy is lost from a propagating

wave. Since the Euler-Bernoulli beam expressed by Eq. (1) is conservative, an energy-conservation relation can be written as

$$\frac{\partial \xi}{\partial t}+\frac{\partial I_e}{\partial x}=0, \tag{4}$$

in which ξ is the wave energy per unit length given by

$$\xi=\frac{1}{2}\rho_e A_e\left(\frac{\partial w_e}{\partial t}\right)^2+\frac{1}{2}E_e J_e\left(\frac{\partial^2 w_e}{\partial x^2}\right)^2, \tag{5}$$

and I_e is the wave intensity given by

$$I_e=-\frac{\partial w_e}{\partial t}Q_e-\frac{\partial^2 w_e}{\partial x \partial t}M_e, \tag{6}$$

with

$$Q_e=-\frac{\partial}{\partial x}\left(E_e J_e\frac{\partial^2 w_e}{\partial x^2}\right), \tag{7a}$$

$$M_e=E_e J_e\frac{\partial^2 w_e}{\partial x^2}, \tag{7b}$$

being the shear force and bending moment within the beam, respectively. If only propagated waves presented in Eq. (2) are taken into account, and applying the real part of Eq. (2) into Eqs. (5) and (6), one can obtain

$$\xi=\frac{1}{2}\rho_e A_e \omega^2 \hat{w}_e{}^2, \tag{8}$$

$$I_e=\omega k^3\left|\hat{w}_e\right|^2 E_e J_e, \tag{9}$$

from which it can be easily found that Eq. (4) is satisfied automatically, and the first term in the left hand side of Eq. (4) represents the wave energy invariance with time per unit length and the second term in the left hand side of Eq. (4) represents the wave intensity invariance along the beam. Thus, the wave amplitude can be calculated by

$$\hat{w}_e = \frac{I_{in}}{\left(\rho_e A_e\right)^{\frac{3}{8}} \left(E_e J_e\right)^{\frac{1}{8}}}, \tag{10}$$

with I_{in} being the incident wave intensity. The strain gain is defined as the ratio between the normal strain amplitude on the GRIN beam and the reference normal strain amplitude on the corresponding location of the background beam as

$$G_s = \frac{\left(\rho_e^{(b)} A_e^{(b)}\right)^{\frac{3}{8}} \left(E_e^{(b)} J_e^{(b)}\right)^{\frac{1}{8}}}{\left(\rho_e A_e\right)^{\frac{3}{8}} \left(E_e J_e\right)^{\frac{1}{8}}} n_e^2, \tag{11}$$

where $n_e = \frac{k}{k_0}$ with k_0 being the wavenumber on the background beam. If the mass density per unit length is unchanged along the GRIN beam, and is equal to that in the reference host beam, Eq. (11) becomes

$$G_s = n_e^{\frac{5}{2}}. \tag{12}$$

From Eq. (12), the strain amplification can be evaluated with a given refractive index. It should be noted that the strain amplifications can be realized with any kinds of slowly varying refractive index profiles, not just the linear one used in the manuscript.

2. Theoretical modeling of the adaptive metamaterial beam with shunted piezoelectric patches

Figure S1 shows the unit cell design of the adaptive metamaterial beam for flexural wave signal amplification which is comprised of a beam with a perfectly bonded piezoelectric patch controlled by a shunting circuit. As shown in the figure, the length and thickness of the beam and the piezoelectric patch are denoted as L_b, h, L_p and h_p, respectively. Z_{sh} represents the impedance of the shunting circuit.

2.1. Governing equations of the adaptive metamaterial beam

For a piezoelectric material, the constitutive relations can be written as

$$S_i = s_{ij}^E T_j + d_{ki} E_k \text{ , } i,j = 1, 2 \ldots 6, k = 1, 2, 3, \tag{13a}$$

$$D_i = d_{ij} T_j + \varepsilon_{ik}^T E_k \text{ , } i,k = 1, 2, 3, j = 1, 2 \ldots 6, \tag{13b}$$

where S_i, T_j, D_i and E_k represent the strain, stress, electric displacement and electric field tensors, respectively. $s_{ij}{}^E$ is the compliance coefficient tensor at constant electric field, ε_{ik}^T is the dielectric constant tensor at constant stress, and d_{ij} is the piezoelectric constant tensor.

In the theoretical model, we ignore variation in the y-direction and implement a plane stress assumption with $T_2 = 0$. The width of the beam is suppressed in all equations. For thin beams, we do not consider shear stresses and strains in the final governing equations, and the piezoelectric constitutive relations for shear strains and shear stresses are ignored. Since the bottom and upper surfaces of the adaptive metamaterial beam are traction free, the normal stress along the z-direction would be extremely small and can thus be ignored. The piezoelectric materials are polarized and

bonded with electrodes along the *z*-direction, thus the assumption $D_1 = D_2 = 0$ and $E_1 = E_2 = 0$ can be used. Therefore, Eqs. (13a) and (13b) can be reduced to

$$S_1 = s_{11}^E T_1 + d_{31} E_3 , \tag{14a}$$

$$D_3 = d_{31} T_1 + \varepsilon_{33}^T E_3 . \tag{14b}$$

For a beam section bonded with a piezoelectric patch, the displacement along *x*-direction is assumed to be linearly distributed in the *z*-direction as

$$u(x,z) = u_0(x) - z\frac{\partial w}{\partial x} , \tag{15}$$

where u_0 and w denote the in-plane and out-of-plane displacements at the mid-plane of the beam ($z = 0$), respectively. When the thickness of the piezoelectric patch is comparable to the thickness of the beam (i.e. the geometry used in the proposed design), the assumption of a constant electric field along the thickness direction is not valid. Therefore, for simplicity and accuracy, a linearly distributed electric field along the thickness direction is assumed in the proposed theoretical model as

$$E_3(x,z) = z \cdot a(x) + b(x) , \tag{16}$$

where functions a and b can be determined with 3D piezoelectric governing equations and corresponding electrical boundary conditions. By considering the kinetic relations and substituting Eqs. (15) and (16) into Eqs. (14a) and (14b), the normal strain and stress along with the electric displacement of the piezoelectric patch can be found as

$$S_1 = \frac{\partial u_0}{\partial x} - z\frac{\partial^2 w}{\partial x^2} , \tag{17a}$$

$$T_1 = \frac{1}{s_{11}^E}\frac{\partial u_0}{\partial x} - \frac{d_{31}}{s_{11}^E} b - z\left(\frac{1}{s_{11}^E}\frac{\partial^2 w}{\partial x^2} + \frac{d_{31}}{s_{11}^E} a\right), \tag{17b}$$

$$D_3 = \frac{d_{31}}{s_{11}^E}\frac{\partial u_0}{\partial x} - \frac{d_{31}^2}{s_{11}^E} b + \varepsilon_{33}^T b - z\left(\frac{d_{31}}{s_{11}^E}\frac{\partial^2 w}{\partial x^2} + \frac{d_{31}^2}{s_{11}^E} a - \varepsilon_{33}^T a\right). \tag{17c}$$

The electrical charge equation of the piezoelectric patch can be written as

$$D_{i,i} = 0. \tag{18}$$

By considering the assumptions for the electrical portion mentioned above ($D_1 = D_2 = 0$), one can obtain

$$\frac{\partial D_3}{\partial z} = 0, \tag{19}$$

and therefore

$$a = \frac{d_{31}}{s_{11}^E \varepsilon_{33}^T - d_{31}^2}\frac{\partial^2 w}{\partial x^2}. \tag{20}$$

As illustrated in Fig. S1, the lower electrode of the piezoelectric patch is grounded, and the upper electrode of the piezoelectric patch is connected to a shunting circuit with the impedance being Z_{sh}, which yields

$$V\left(z = \frac{h}{2}\right) = 0, \tag{21a}$$

$$V\left(z = \frac{h}{2} + h_p\right) = I \cdot Z_{sh}, \tag{21b}$$

where V is the electric potential in the piezoelectric patch, and I is the current flowing through the shunting circuit. The relationship between the electric field and potential in the piezoelectric patch can be expressed as

$$E_3 = -\frac{\partial V}{\partial z} . \tag{22}$$

The potential can then be obtained through the integration of Eq. (16) as

$$V = -\left(\frac{a}{2} z^2 + bz + c \right), \tag{23}$$

where c is a function determined by considering the electrical boundary condition in Eq. (21a) as

$$c = -\frac{a}{8} h^2 - \frac{b}{2} h . \tag{24}$$

The electrical current and the corresponding free charge on the upper electrode of the piezoelectric patch Q are related by

$$I = \frac{\partial Q}{\partial t} , \tag{25}$$

in which the free charge is calculated as

$$Q = \int_{x_1}^{x_1 + L_p} D_3 dx , \tag{26}$$

with x_1 being the coordinate at the left edge of the piezoelectric patch. By combining Eqs. (17c), (20), (21b) and Eqs. (23) – (26), the potential on the upper electrode and function b can be written as

$$A = \frac{i\omega \frac{d_{31} Z_{sh}}{s_{11}^E} \int_{x_1}^{x_1+L_p} \left(\frac{\partial u_0}{\partial x} - \frac{h+h_p}{2} \frac{\partial^2 w}{\partial x^2} \right) dx}{1 + i\omega C_p^T \left(1 - k_{31}^2\right) Z_{sh}}, \tag{27}$$

$$b = -\frac{A}{h_p} - \frac{a}{2}\left(h + h_p\right), \tag{28}$$

where $k_{31}^2 = \frac{d_{31}^2}{s_{11}^E \varepsilon_{33}^T}$. According to Euler-Bernoulli beam theory, the bending moment and normal force in the beam section with a piezoelectric patch are

$$M = \int_{-\frac{h}{2}}^{\frac{h}{2}} -T_1^{(b)} z dz + \int_{\frac{h}{2}}^{\frac{h}{2}+h_p} -T_1 z dz, \tag{29a}$$

$$N = \int_{-\frac{h}{2}}^{\frac{h}{2}} T_1^{(b)} dz + \int_{\frac{h}{2}}^{\frac{h}{2}+h_p} T_1 dz. \tag{29b}$$

Substituting Eq. (17b) into Eqs. (29a) and (29b), we can derived the relations

$$M = I \frac{\partial^2 w}{\partial x^2} - J \frac{\partial u_0}{\partial x} - FA, \tag{30a}$$

$$N = K \frac{\partial u_0}{\partial x} - J \frac{\partial^2 w}{\partial x^2} + GA. \tag{30b}$$

in which

$$I = \frac{Eh^3}{12} + \frac{6hh_p^2 + 3h^2 h_p + 4h_p^3}{12 s_{11}^E} + \frac{h_p^3 d_{31}^2}{12 s_{11}^E \left(s_{11}^E \varepsilon_{33}^T - d_{31}^2 \right)},$$

$$J = \frac{h_p^2 + hh_p}{2 s_{11}^E},$$

$$K = Eh + \frac{h_p}{s_{11}^E},$$

$$F = \frac{\left(h + h_p\right) d_{31}}{2 s_{11}^E},$$

$$G = \frac{d_{31}}{s_{11}^E},$$

where E is the Young's modulus of the beam. The shear force can then be calculated by

$$T = I \frac{\partial^3 w}{\partial x^3} - J \frac{\partial^2 u_0}{\partial x^2}. \tag{31}$$

Based on the assumptions made above, the equations of motion for flexural and longitudinal waves on the beam section with a piezoelectric patch can be written as

$$\frac{\partial^2 M}{\partial x^2} + \bar{\rho} \frac{\partial^2 w}{\partial t^2} = 0, \tag{32a}$$

$$\frac{\partial N}{\partial x} - \bar{\rho} \frac{\partial^2 u_0}{\partial t^2} = 0, \tag{32b}$$

where $\bar{\rho} = \rho_b h + \rho_p h_p$ with ρ_b and ρ_p being the mass density of the beam and the piezoelectric patch, respectively. Therefore by substituting Eqs. (30a) and (30b) into Eqs. (32a) and (32b), the final coupled governing equations for the beam section with a piezoelectric patch become

$$I \frac{\partial^4 w}{\partial x^4} - J \frac{\partial^3 u_0}{\partial x^3} + \bar{\rho} \frac{\partial^2 w}{\partial t^2} = 0, \tag{33a}$$

$$K \frac{\partial^2 u_0}{\partial x^2} - J \frac{\partial^3 w}{\partial x^3} - \bar{\rho} \frac{\partial^2 u_0}{\partial t^2} = 0. \tag{33b}$$

For a beam section without a piezoelectric patch, the uncoupled governing equations for flexural and longitudinal waves can be written as

$$I^{(b)} \frac{\partial^4 w^{(b)}}{\partial x^4} + \rho^{(b)} \frac{\partial^2 w^{(b)}}{\partial t^2} = 0\,, \tag{34a}$$

$$K^{(b)} \frac{\partial^2 u_0^{(b)}}{\partial x^2} - \rho^{(b)} \frac{\partial^2 u_0^{(b)}}{\partial t^2} = 0\,, \tag{34b}$$

where

$$I^{(b)} = \frac{Eh^3}{12}\,,$$

$$K^{(b)} = Eh\,,$$

$$\rho^{(b)} = \rho_b h\,,$$

and $w^{(b)}$ and $u_0^{(b)}$ are the out-of-plane and in-plane displacement on the mid-plane of the beam without the piezoelectric patch, respectively.

2.2. Dispersion relations of the adaptive metamaterial beam with uniform circuits

To calculate the dispersion relations of the adaptive metamaterial beam with uniform circuits, the harmonic wave solutions of Eqs. (33a) and (33b) are assumed as

$$w = \bar{A} e^{i\omega t + \lambda x}\,, \tag{35a}$$

$$u_0 = \bar{B} e^{i\omega t + \lambda x}\,. \tag{35b}$$

By applying Eqs. (35a) and (35b) to Eqs. (33a) and (33b), a characteristic equation can be obtained as

$$\left(IK - J^2\right)\lambda^6 + I\omega^2\bar{\rho}\lambda^4 - K\omega^2\bar{\rho}\lambda^2 - \omega^4\bar{\rho}^2 = 0 . \tag{36}$$

Therefore, the general solutions of Eqs. (33a) and (33b) can be expressed as

$$w = \sum_{n=1}^{6} \bar{A}_n e^{\lambda_n x} , \tag{37a}$$

$$u_0 = \sum_{n=1}^{6} \bar{A}_n \alpha_n e^{\lambda_n x} , \tag{37b}$$

where λ_n are the six roots of Eq. (36), $\bar{A}_n$ are arbitrary constants, and $\alpha_n = \dfrac{I\lambda_n^4 - \omega^2\bar{\rho}}{J\lambda_n^3}$. All time harmonic terms are suppressed from equations. In addition, with Eqs. (37a) and (37b), the electrical potential on the upper electrode of the piezoelectric patch can be calculated as

$$A = \sum_{n=1}^{6} \bar{A}_n \beta_n e^{\lambda_n x} , \tag{38}$$

in which

$$\beta_n = \frac{i\omega \dfrac{d_{31} Z_{sh}}{s_{11}^E} \left(\alpha_n - \dfrac{h + h_p}{2} \lambda_n \right) \left(e^{\lambda_n L_p} - 1 \right) e^{\lambda_n (x_1 - x)}}{1 + i\omega C_p^T \left(1 - k_{31}^2 \right) Z_{sh}} . \tag{39}$$

The general solution of Eqs. (34a) and (34b) can be written as

$$w^{(b)} = \sum_{n=1}^{4} \bar{A}_n^{(b)} e^{\lambda_n^{(b)} x} , \tag{40a}$$

$$u_0^{(b)} = \sum_{n=5}^{6} \bar{A}_n^{(b)} e^{\lambda_n^{(b)} x} , \tag{40b}$$

where $\bar{A}_n^{(b)}$ are arbitrary constants, and

$$\lambda_{1,2}^{(b)} = \pm i\sqrt[4]{\rho^{(b)}\omega^2 / I^{(b)}} ,$$

$$\lambda_{3,4}^{(b)} = \pm\sqrt[4]{\rho^{(b)}\omega^2 / I^{(b)}} ,$$

$$\lambda_{5,6}^{(b)} = \pm i\sqrt{\rho^{(b)}\omega^2 / E} .$$

The dispersion relations of the adaptive metamaterial beam can be solved by using the transfer matrix method. For the beam section with a piezoelectric patch, applying the general solutions (Eqs. (37a) and (37b)) into Eqs. (30a), (30b) and (31), one can find

$$\mathbf{Y_p} = \mathbf{B_p}\mathbf{V_p} , \tag{41}$$

where

$$\mathbf{Y_p} = \begin{bmatrix} w & \frac{\partial w}{\partial x} & M & T & u_0 & N \end{bmatrix}^{\mathrm{T}} ,$$

$$\mathbf{V_p} = \begin{bmatrix} \bar{A}_1 e^{\lambda_1 x} & \bar{A}_2 e^{\lambda_2 x} & \bar{A}_3 e^{\lambda_3 x} & \bar{A}_4 e^{\lambda_4 x} & \bar{A}_5 e^{\lambda_5 x} & \bar{A}_6 e^{\lambda_6 x} \end{bmatrix}^{\mathrm{T}} ,$$

$$\mathbf{B_p} = \begin{bmatrix} a_{11} & a_{12} & a_{13} & a_{14} & a_{15} & a_{16} \\ a_{21} & a_{22} & a_{23} & a_{24} & a_{25} & a_{26} \\ a_{31} & a_{32} & a_{33} & a_{34} & a_{35} & a_{36} \\ a_{41} & a_{42} & a_{43} & a_{44} & a_{45} & a_{46} \\ a_{51} & a_{52} & a_{53} & a_{54} & a_{55} & a_{56} \\ a_{61} & a_{62} & a_{63} & a_{64} & a_{65} & a_{66} \end{bmatrix},$$

with

$$a_{1n} = 1,$$

$$a_{2n} = \lambda_n ,$$

$$a_{3n} = I\lambda_n^2 - J\alpha_n\lambda_n - F\beta_n,$$

$$a_{4n} = I\lambda_n^3 - J\alpha_n\lambda_n^2,$$

$$a_{5n} = \alpha_n,$$

$$a_{6n} = K\alpha_n\lambda_n - J\lambda_n^2 + G\beta_n.$$

Eq. (41) at the two edges of the piezoelectric patch can be written as

$$\mathbf{Y_p}\left(x = x_1\right) = \mathbf{B_p}\left(x = x_1\right)\mathbf{V_p}\left(x = x_1\right), \tag{42a}$$

$$\mathbf{Y_p}\left(x = x_1 + L_p\right) = \mathbf{B_p}\left(x = x_1 + L_p\right)\mathbf{V_p}\left(x = x_1 + L_p\right). \tag{42b}$$

The harmonic wave solutions indicate

$$\mathbf{V_p}\left(x = x_1 + L_p\right) = \mathbf{D_p}\mathbf{V_p}\left(x = x_1\right), \tag{43}$$

where

$$\mathbf{D_p} = \begin{bmatrix} e^{\lambda_1 L_p} & 0 & 0 & 0 & 0 & 0 \\ 0 & e^{\lambda_2 L_p} & 0 & 0 & 0 & 0 \\ 0 & 0 & e^{\lambda_3 L_p} & 0 & 0 & 0 \\ 0 & 0 & 0 & e^{\lambda_4 L_p} & 0 & 0 \\ 0 & 0 & 0 & 0 & e^{\lambda_5 L_p} & 0 \\ 0 & 0 & 0 & 0 & 0 & e^{\lambda_6 L_p} \end{bmatrix}.$$

Combining Eqs. (42a), (42b) and (43), the relationship between the left and right edges of the piezoelectric patch can be found as

$$\mathbf{Y_p}\left(x = x_1 + L_p\right) = \mathbf{B_p}\left(x = x_1 + L_p\right)\mathbf{D_p}\mathbf{B_p^{-1}}\left(x = x_1 + L_p\right)\mathbf{Y_p}\left(x = x_1\right). \tag{44}$$

Similarly, the relationship between the left and right edges of the beam without a piezoelectric patch can also be obtained as

$$\mathbf{Y_b}\left(x = x_1 + L_b\right) = \mathbf{B_b}\mathbf{D_b}\mathbf{B_b^{-1}}\mathbf{Y_b}\left(x = x_1 + L_p\right), \tag{45}$$

with

$$\mathbf{Y_b} = \left[w^{(b)} \;\; \frac{\partial w^{(b)}}{\partial x} \;\; M^{(b)} \;\; T^{(b)} \;\; u_0^{(b)} \;\; N^{(b)} \right]^{\mathrm{T}},$$

$$\mathbf{B_b} = \begin{bmatrix} 1 & 1 & 1 & 1 & 0 & 0 \\ \lambda_1^{(b)} & \lambda_2^{(b)} & \lambda_3^{(b)} & \lambda_4^{(b)} & 0 & 0 \\ I^{(b)}\left(\lambda_1^{(b)}\right)^2 & I^{(b)}\left(\lambda_2^{(b)}\right)^2 & I^{(b)}\left(\lambda_3^{(b)}\right)^2 & I^{(b)}\left(\lambda_4^{(b)}\right)^2 & 0 & 0 \\ I^{(b)}\left(\lambda_1^{(b)}\right)^3 & I^{(b)}\left(\lambda_2^{(b)}\right)^3 & I^{(b)}\left(\lambda_3^{(b)}\right)^3 & I^{(b)}\left(\lambda_4^{(b)}\right)^3 & 0 & 0 \\ 0 & 0 & 0 & 0 & 1 & 1 \\ 0 & 0 & 0 & 0 & K^{(b)}\lambda_5^{(b)} & K^{(b)}\lambda_6^{(b)} \end{bmatrix},$$

$$\mathbf{D_b} = \begin{bmatrix} e^{\lambda_1^{(b)}\left(L_b - L_p\right)} & 0 & 0 & 0 & 0 & 0 \\ 0 & e^{\lambda_2^{(b)}\left(L_b - L_p\right)} & 0 & 0 & 0 & 0 \\ 0 & 0 & e^{\lambda_3^{(b)}\left(L_b - L_p\right)} & 0 & 0 & 0 \\ 0 & 0 & 0 & e^{\lambda_4^{(b)}\left(L_b - L_p\right)} & 0 & 0 \\ 0 & 0 & 0 & 0 & e^{\lambda_5^{(b)}\left(L_b - L_p\right)} & 0 \\ 0 & 0 & 0 & 0 & 0 & e^{\lambda_6^{(b)}\left(L_b - L_p\right)} \end{bmatrix}.$$

The continuity condition at the edge of the piezoelectric patch requires

$$\mathbf{Y_p}\left(x = x_1 + L_p\right) = \mathbf{Y_b}\left(x = x_1 + L_p\right). \tag{46}$$

Combining Eqs. (44) – (46), the transfer matrix for the unit cell presented in Fig. S1 can be established as

$$\mathbf{Y_b}\left(x = x_1 + L_b\right) = \mathbf{T_{pb}}\mathbf{Y_p}\left(x = x_1\right), \tag{47}$$

with

$$\mathbf{T_{pb}} = \mathbf{B_b}\mathbf{D_b}\mathbf{B_b^{-1}}\mathbf{B_p}\left(x = x_1 + L_p\right)\mathbf{D_p}\mathbf{B_p^{-1}}\left(x = x_1 + L_p\right).$$

According to the Bloch theorem, $\mathbf{Y_b}\left(x = x_1 + L_b\right)$ and $\mathbf{Y_p}\left(x = x_1\right)$ are related by

$$\mathbf{Y_b}\left(x = x_1 + L_b\right) = e^{ik_x L_b}\mathbf{Y_p}\left(x = x_1\right), \tag{48}$$

where k_x is the wavenumber along the wave propagation direction. Comparing Eqs. (47) and (48), the dispersion relations can then be calculated from the eigensystem as

$$\mathbf{T_{pb}}\mathbf{Y_p}\left(x = x_1\right) = e^{ik_x L_b}\mathbf{Y_p}\left(x = x_1\right). \tag{49}$$

2.3. Wave propagation along the GRIN adaptive metamaterial beam

Consider the finite GRIN adaptive metamaterial beam shown in Fig. S2 with N number of unit cells. As illustrated in the figure, the n-th unit cell of the adaptive metamaterial beam consists of a host beam bonded with a piezoelectric patch which is shunted by an electric circuit with an equivalent impedance of ${}^{n}Z_{sh}$. In order to characterize the wave propagation properties along the GRIN metamaterial beam, the transfer matrix method will be employed. The host beam is assumed to be infinitely long to suppress the reflected waves from the boundaries. Based on Eqs. (41) and (45), the **Y** matrices at the right hand side boundary of the (n-1)-th unit cell and the left hand side boundary of the n-th unit cell can be written as

$$ {}^{n-1}\mathbf{Y}_{\mathbf{b}}^{(4)} = {}^{n-1}\mathbf{B}_{\mathbf{b}}^{(4)}\, {}^{n-1}\mathbf{V}_{\mathbf{b}}^{(4)}, \tag{50} $$

$$ {}^{n}\mathbf{Y}_{\mathbf{p}}^{(1)} = {}^{n}\mathbf{B}_{\mathbf{p}}^{(1)}\, {}^{n}\mathbf{V}_{\mathbf{p}}^{(1)}, \tag{51} $$

where ${}^{n-1}\mathbf{V}_{\mathbf{b}}^{(4)}$ and ${}^{n}\mathbf{V}_{\mathbf{p}}^{(1)}$, denoted in the figure, are wave amplitudes at the right hand side boundary of the (*n*-1)-th unit cell and the left hand side boundary of the *n*-th unit cell, respectively, and ${}^{n-1}\mathbf{B}_{\mathbf{b}}^{(4)}$ and ${}^{n}\mathbf{B}_{\mathbf{p}}^{(1)}$ are corresponding $\mathbf{B}_{\mathbf{b}}$ and $\mathbf{B}_{\mathbf{p}}$ matrices, respectively. Applying continuity conditions to Eqs. (50) and (51), wave amplitudes at the right hand side boundary of the (*n*-1)-th unit cell can be related to wave amplitudes at the left hand side boundary of the *n*-th unit cell as

$$ {}^{n-1}\mathbf{V}_{\mathbf{b}}^{(4)} = {}^{n-1}\mathbf{B}_{\mathbf{b}}^{\mathbf{-1}(4)}\, {}^{n}\mathbf{B}_{\mathbf{p}}^{(1)}\, {}^{n}\mathbf{V}_{\mathbf{p}}^{(1)}. \tag{52} $$

By following the same procedure in deriving Eqs. (50) – (52), similar wave amplitude relations inside the *n*-th unit cell can be found as

$$ {}^{n}\mathbf{V}_{\mathbf{p}}^{(2)} = {}^{n}\mathbf{B}_{\mathbf{p}}^{\mathbf{-1}(2)}\, {}^{n}\mathbf{B}_{\mathbf{b}}^{(3)}\, {}^{n}\mathbf{V}_{\mathbf{b}}^{(3)}. \tag{53} $$

Combining Eqs. (43), (45), (52) and (53), one can find the wave amplitude relations between the (*n*-1)-th unit cell and the *n*-th unit cell to be

$$ {}^{n-1}\mathbf{V}_{\mathbf{b}}^{(4)} = {}^{n}\mathbf{T}\, {}^{n}\mathbf{V}_{\mathbf{b}}^{(4)}, \tag{54} $$

where

$$ {}^{n}\mathbf{T} = {}^{n-1}\mathbf{B}_{\mathbf{b}}^{\mathbf{-1}(4)}\, {}^{n}\mathbf{B}_{\mathbf{p}}^{(1)}\mathbf{D}_{\mathbf{p}}^{\mathbf{-1}}\, {}^{n}\mathbf{B}_{\mathbf{p}}^{\mathbf{-1}(2)}\, {}^{n}\mathbf{B}_{\mathbf{b}}^{(3)}\mathbf{D}_{\mathbf{b}}^{\mathbf{-1}}. $$

Thus, the final transfer matrix for the GRIN adaptive metamaterial beam is defined by

$$ {}^{0}\mathbf{V}_{\mathbf{b}}^{(4)} = \mathbf{T}\, {}^{N}\mathbf{V}_{\mathbf{b}}^{(4)}, \tag{55} $$

with

$$\mathbf{T} = {}^{1}\mathbf{T}\,{}^{2}\mathbf{T}\,{}^{3}\mathbf{T} \cdots {}^{N-1}\mathbf{T}\,{}^{N}\mathbf{T}.$$

An incident flexural wave propagating in the positive x-direction with an amplitude $A_0^{(+)}$ is assumed at the left hand side boundary of the GRIN adaptive metamaterial beam as

$${}^{0}\mathbf{V}_{\mathbf{b}}^{(4)} = \left[A_0^{(+)}, A_0^{(-)}, 0, B_0^{(-)}, 0, C_0^{(-)} \right], \tag{56}$$

where $A_0^{(-)}$, $B_0^{(-)}$ and $C_0^{(-)}$ denote wave amplitudes of reflected flexural, evanescent and longitudinal waves from the adaptive metamaterial beam to the host beam, respectively. And the boundary conditions at the right hand boundary of the N-th unit cell can be expressed as

$${}^{N}\mathbf{V}_{\mathbf{b}}^{(4)} = \left[A_N^{(+)}, 0, B_N^{(+)}, 0, C_N^{(+)}, 0 \right], \tag{57}$$

where $A_N^{(+)}$, $B_N^{(+)}$ and $C_N^{(+)}$ denote wave amplitudes of transmitted flexural, evanescent and longitudinal waves from the adaptive metamaterial beam to the host beam, respectively. Thus, based on Eq. (57), all the transmitted wave amplitudes can be solved by using the first, third and fifth equations in Eq. (55), and the final wave fields along the GRIN adaptive metamaterial beam can be retrieved by Eq. (55).

2.4. Voltage gain calculations

According to Eq. (27), the potential on the upper electrode of the PZT patch with an open circuit can be obtained by setting the shunting impedance to be infinity in the form

$$A_{open} = \frac{i\omega \dfrac{d_{31}}{s_{11}^{E}} \displaystyle\int_{x_1}^{x_1+L_p} \left(\frac{\partial \overline{u}_0}{\partial x} - \frac{h+h_p}{2} \frac{\partial^2 \overline{w}}{\partial x^2} \right) dx}{i\omega C_p^{T} \left(1-k_{31}^{2}\right)}, \tag{58}$$

where $\bar{u}_0$ and $\bar{w}$ represent the in-plane and out-of-plane displacements on the mid-plane of the beam (z = 0) with an open circuit, respectively. The voltage gain, which is defined by the ratio between the potential amplitude with the shunting circuit and the potential amplitude with open circuit on the upper electrode of the PZT patch as

$$G_V = \frac{G_s}{1 + \dfrac{1}{i\omega C_p^T \left(1 - k_{31}^2\right) Z_{sh}}}, \tag{59}$$

in which $G_s = \dfrac{\int_{x_1}^{x_1+L_p} \left(\dfrac{\partial u_0}{\partial x} - \dfrac{h+h_p}{2} \dfrac{\partial^2 w}{\partial x^2} \right) dx}{\int_{x_1}^{x_1+L_p} \left(\dfrac{\partial \bar{u}_0}{\partial x} - \dfrac{h+h_p}{2} \dfrac{\partial^2 \bar{w}}{\partial x^2} \right) dx}$ is the averaged strain ratio (gain) of the PZT patch between shunting circuit and open circuit conditions.

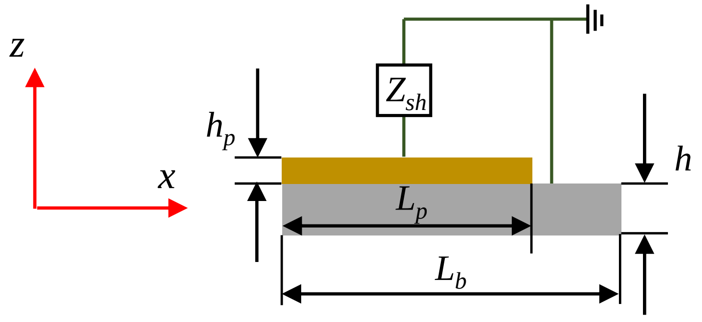

Fig. S1 Unit cell design of the adaptive metamaterial beam.

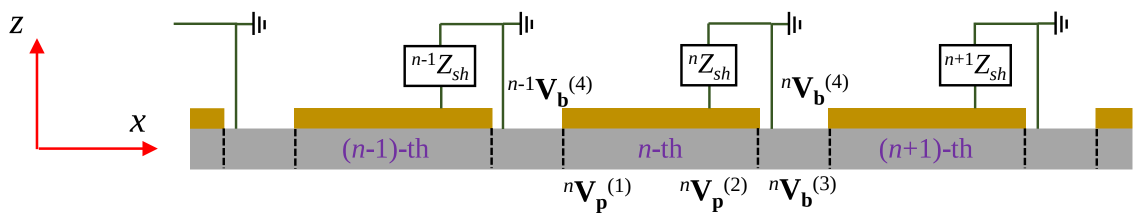

Fig. S2 Schematic of the adaptive GRIN metamaterial beam.
